# The clinical value of preoperative 3D planning and 3D surgical guides for Imhäuser osteotomy in slipped capital femoral epipysis: a retrospective study

**DOI:** 10.1186/s41205-024-00205-2

**Published:** 2024-03-01

**Authors:** Vera Lagerburg, Michelle van den Boorn, Sigrid Vorrink, Ihsane Amajjar, Melinda M. E. H. Witbreuk

**Affiliations:** 1 Medical Physics, OLVG, Amsterdam, The Netherlands; 2 Department of Medical Technology, OLVG, Amsterdam, The Netherlands; 3 Department of Orthopedic Surgery, OLVG, Amsterdam, The Netherlands

**Keywords:** 3D printed surgical guide, 3D planning, Slipped capital femoral epiphysis, Imhäuser osteotomy

## Abstract

**Background:**

Accurate repositioning of the femoral head in patients with Slipped Capital Femoral Epiphysis (SCFE) undergoing Imhäuser osteotomy is very challenging. The objective of this study is to determine if preoperative 3D planning and a 3D-printed surgical guide improve the accuracy of the placement of the femoral head.

**Methods:**

This retrospective study compared outcome parameters of patients who underwent a classic Imhäuser osteotomy from 2009 to 2013 with those who underwent an Imhäuser osteotomy using 3D preoperative planning and 3D-printed surgical guides from 2014 to 2021. The primary endpoint was improvement in Range of Motion (ROM) of the hip. Secondary outcomes were radiographic improvement (Southwick angle), patient-reported clinical outcomes regarding hip and psychosocial complaints assessed with two questionnaires and duration of surgery.

**Results:**

In the 14 patients of the 3D group radiographic improvement was slightly greater and duration of surgery was slightly shorter than in the 7 patients of the classis Imhäuser group. No difference was found in the ROM, and patient reported clinical outcomes were slightly less favourable.

**Conclusions:**

Surprisingly we didn’t find a significant difference between the two groups. Further research on the use of 3D planning an 3D-printed surgical guides is needed.

**Trial registration:**

Approval for this study was obtained of the local ethics committees of both hospitals.

## Background

**Fig. 1:**
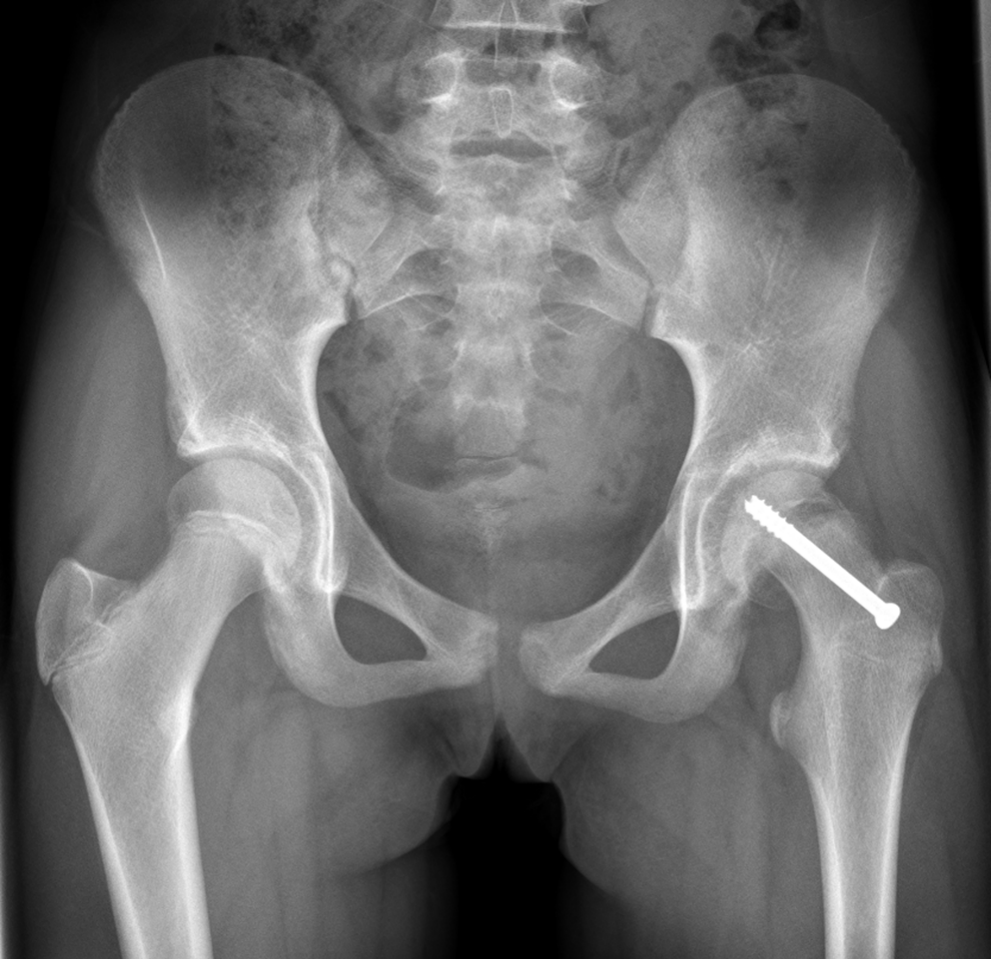
X-ray of a patient with on the left of the image the healthy hip and on the right the affected hip with a pin in-situ

Accurate repositioning of the femur head in patients with Slipped Capital Femoral Epiphysis (SCFE) undergoing Imhäuser osteotomy is very challenging. In adolescents, SCFE is the most common hip disorder [1]. In SCFE, the femoral neck slips from the femoral head at the site of the epiphysis. Specifically, the proximal femoral neck and shaft move forward and rotate outward while the femoral head remains in the acetabulum [2]. The slippage typically takes place in adolescence. The cause is still unknown, either the force on the oblique situated growth plate is too high, or a normal force on a weak physis can cause the slip. Most likely it is a combination of these two. Important complications that can occur in patients with SCFE are increasing displacement of the femur neck, avascular necrosis of the femur head and chondrolysis [3]. Diagnosis of SCFE is done based on a combination of physical examination and X-rays. The severity of SCFE is evaluated using the Southwick slip angle [4].. SCFE can be classified into two subtypes based on the stability of the physis: stable and unstable. The stable type refers to patients who can bear weight, while the unstable type refers to those who are unable to walk [5]. Surgical intervention is always needed to prevent further slippage. In the Netherlands, 11.6 per 100.000 children are treated surgically for SCFE each year [2]. Currently, the golden standard for treatment is in-situ fixation (epiphysiodesis) (Fig. 1). Patients with a limited range of motion often require an osteotomy. The percentage of patients operated on SCFE differs among hospitals. In case of a CAM lesion, which is caused by the metaphyseal hump, the femoroacetabular impingement could be reduced by positioning the CAM lesion of the metaphysis further aside from the acetabular rim. The long-term goal is to diminish or delay the prevalence coxarthrosis later in life [6–9].

The Imhäuser osteotomy is planned with the combination of radiographical imaging (X-rays and/or CT scans) and physical examination. Regardless of the careful preoperative planning, the surgery is challenging to execute. The exact placement of the cutting blade is hindered by the small operation incision and subcutaneous tissues and therefore limited view on the femur. During the procedure, a wedge is removed from the femur. After removal of this wedge, the head of the femur is rotated and translated and placed in the best possible position. Without a reference it is challenging to determine the correct orientation of the head of the femur relative to the distal part of the femur.

The difficulty of the accurate positioning of the femur head can result in limited clinical improvement, and consequently of diminished mobility. Longer surgery time and extended use of fluoroscopy perioperatively are also due to the difficulty of the surgery. Both could mean a higher risk of infection, a higher radiation dosage and higher financial costs. Also, there is the risk of more chronic complaints requiring closer monitoring. A more accurate and time-efficient procedure is necessary to improve clinical results and diminish operation risks.

3D preoperative planning is one of the prospects that could be a solution for the challenging procedure. Since the emergence of 3D printing, it has been widely applied within multiple fields in healthcare such as cranio-maxillofacial surgery, anesthesiology and neurosurgery. Multiple studies have been conducted in those fields to assess the added value of 3D printing, varying from improved accuracy of surgeries and reduced costs to improved training skills of surgeons [10–16]. Within orthopedic surgery, 3D printing also is used for several indications, one of which is SCFE [17]. However, evidence for the added value of 3D-printed surgical guides for SCFE is scarce [18]. Cherkasskiy et al. [17] showed that the use of 3D patient specific models resulted in shorter OR times and less fluoroscopy dose (although not statistically significant) while maintaining the same surgical results. Zakani et al. [18] performed the correction osteotomy on 3D-printed bones and found an improved accuracy of the guided group compared to the non-guided group. The guided surgery group also required significantly less drilling time and intraoperative X-rays.

In our institute, the Imhäuser procedure is 3D planned before the surgery, and surgical guides are custom-made to fit each patient individually to assist the surgeon with the correct placement of the cutting blade and accurate placement of the head of the femur during the surgery. In this study we examine the clinical and radiographical differences between patients who had the classic Imhäuser procedure, versus patients whose surgery was 3D planned, including the use of surgical guides.

The main goal of this study was to determine if the use of 3D preoperative planning and 3D-printed guides resulted in better patient outcomes than the classic Imhäuser osteotomy, defined as improvement of the Range of Motion of the hip.

## Methods and patient population

In this study all patients were included who underwent an Imhäuser osteotomy in two hospitals between 2009 and 2021 and gave informed consent. The indication of such an operation was limited internal rotation of less than 20 degrees of the affected hip. The surgery is performed only in patients with persistent complaints (pain, discomfort), or patients with severely limited ROM (Range of Motion).

All patients were operated by the same orthopedic surgeon. Approval was obtained of the local ethics committees of both hospitals.

### Study design

Patients who were operated on between 2009 and 2013 underwent the classic Imhäuser procedure [19]. The orthopedic surgeon performed a preoperative planning based on the pre-operative radiographs and physical examination. The surgery was performed ‘judge by eye’.

**Fig. 2:**
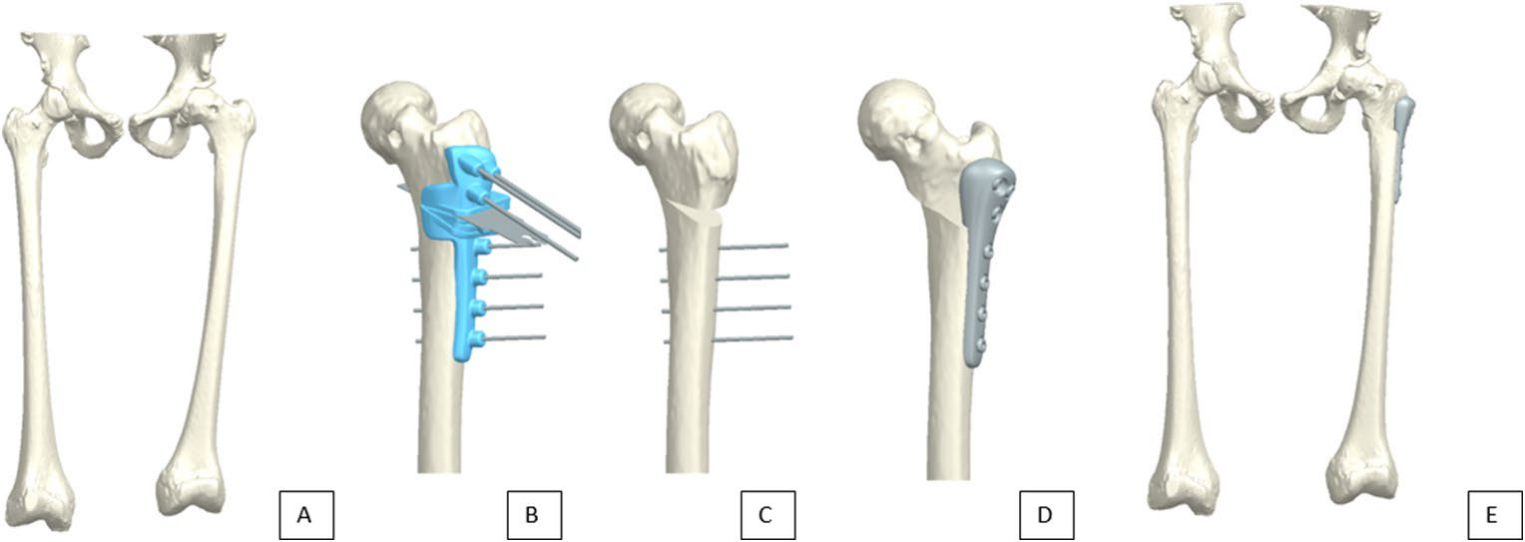
(**a**) 3D segmented model of a healthy (left) and affected femur of a patient, (**b**) the affected femur and its surgical guide, (**c**) the affected femur with the k-wires in place and the wedge removed, (**d**) the affected femur with a titanium plate positioned and (**e**) 3D segmented model of a healthy (left) and operated femur with the plate in place

Patients who were operated between 2014 and 2021 underwent an Imhäuser osteotomy based on a 3D preoperative planning and a surgical guide. A low-dose CT scan was made of the affected and healthy femur. The healthy side was used for planning the osteotomy. If both sides were affected, the planning was performed based on reference patients with the same age and weight in combination with information from literature [20, 21]. The CT scan was segmented using the computer software D2P, 3D systems. A preoperative planning was performed by mirroring the healthy side onto the affected side. The ideal cut was determined based on the individual patient anatomy in combination with several predetermined design criteria, e.g. the head of the femur and the femur steel should have at least 50% contact after surgery. Based on this planning and the commercially available plate a surgical guide was designed using Solidworks, Dassault Systèmes, to fit on the affected femur (Fig. 2). The guides were designed to directly fit the 3D bone model generated from the segmentation.he surgical guide was printed with an EOS p100 printer in nylon PA12 and sterilized to be used during surgery. The surgeon exposed the femur and applied the 3D-printed guide onto the femur. First the position of the screws was determined by placing k-wires through the surgical guide and after this had been checked with fluoroscopy the femur was sawed through the surgical guide. Subsequently, the femur head was rotated and translated and a patient-specific plate was used to fixate the femur in the new position (Fig. 2).

### Outcome assessment

The primary endpoint of the study was improvement in Range of Motion (ROM) of the hip. Secondary outcomes were radiographic improvement (Southwick angle), patient-reported clinical outcomes assessed with two questionnaires and duration of surgery.

Improvement in ROM of the hip was defined as the difference in the ROM, specifically flexion, internal rotation, external rotation and abduction of the hip, before and after surgery, and compared to normal values. We assumed the following normal values of the hip: Flexion Extension: 130-0-0; Internal External rotation: 45-0-45; Abduction Adduction: 40-0-40 [20, 21].

**Fig. 3:**
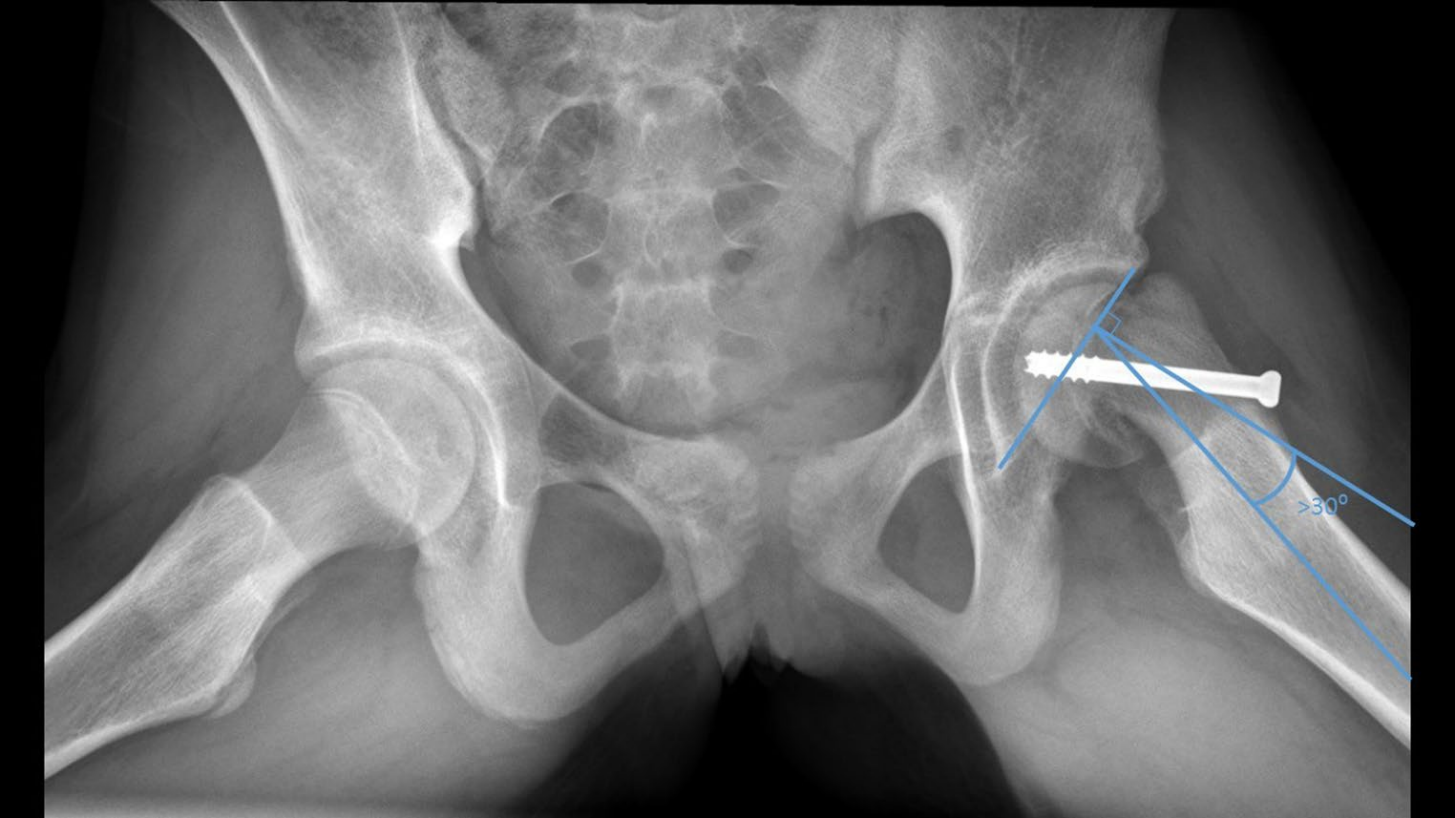
Example of an X-ray with the measurement of the Southwick angle

Differences in radiographic improvements were measured by comparing pre- and postoperative X-rays of patients, specifically the Southwick angle, which gives an indication of the severity of SCFE. Mild SCFE is classified at < 30°, moderate is 30°-50°, severe is > 50° [22]. The Southwick angle is a radiographic angle used to measure the severity of a slipped capital femoral epiphysis (SCFE) on a radiograph (Fig. 3) [23]. The angle is measured on a frog lateral view of the bilateral hips. It is measured by drawing a line perpendicular to a line connecting two points at the posterior and anterior tips of the epiphysis at the physis. A third line is drawn down the axis of femur. The angle between the perpendicular line and the femoral shaft line is the angle The Southwick angle was assessed on the Lauenstein view and measured 3 times by the same orthopedic surgeon. The average of the different measurements was used for the assessment of the differences.

Patients were sent two questionnaires: Hip disability and Osteoarthritis Outcome Score- Physical function Short form (HOOS-PS) and the Pediatric Quality of Life inventory (PedsQL), either electronically or by mail. The HOOS-PS examines hip-related complaints where a higher score means more complaints. The PedsQL examines more aspects of the patient’s life such as psychosocial complaints, where a lower score means more complaints. Both questionnaires have a score ranging from 0 to 100. The electronic database Castor EDC was used to store the results of the questionnaires.

Duration of surgery was extracted from the Electronic Patient Record.

### Statistical analysis

SPSS version 27 was used for the statistical analysis. Normal distribution was tested. T-tests were used for normally distributed data, while Man-Whitney U tests were applied to non parametric distributed data. Improvement in ROM of the hip, improvement in Southwick angle and both patient questionnaires were compared between the two groups.

Duration of follow-up was seen as a potential confounder in ROM and the scores in the questionnaires. Therefore, linear regression models were used to examine the potential confounder. Linear regression models were also used to examine a potential learning curve.

## Results

In total, 24 children and adolescents with SCFE were operated between 2009 and 2021, of which 21 were included in this study. One patient didn’t react on our call and from two patients we didn’t have recent contact details. Table 1 shows the patient characteristics, Table 2 shows the differences in ROM before and after surgery and Table 3 shows the differences in ROM after surgery, compared to the normal values, the radiographical outcome and the outcome of patient questionnaires.

**Table 1:** Patient characteristics

Characteristics	Classic Imhäuser group (*N* = 7)	3D planned Imhäuser group (*N* = 14)	P value
Sex, female, n (%)	3 (42,9%)	10 (71,4%)	0,22
Age at surgery (mean ± SD)	14,0 ± 2,8	13,6 ± 1,8	0,68
BMI (mean ± SD)	26,5 ± 4,9	24,0 ± 6,6	0,42
Southwick angle preoperatively (mean ± SD)	52,6 ± 16,7	58,8 ± 13,4	0,37
Mean follow up period for ROM in days (mean ± SD)	33,4 ± 20,6	17,7 ± 10,1	0,03
Mean follow up period for questionnaires in months (mean ± SD)	109,9 ± 22,7	30,2 ± 20,6 *	< 0,001

*Significant difference between the two groups

Clinical relevant improvement in ROM after surgery was observed in both groups (Table 2). However, no statistically significant difference was found between the classic Imhäuser group and the 3D planned group. Table 3 shows the clinical results, showing primary and secondary outcomes for the 21 participants. The ROM was compared with the normal values. No statistically significant difference was found in the ROM between the classic Imhäuser group and the 3D planned group.

**Table 2:** Clinical results showing Range Of Motion preoperative, postoperative and improvement, defined as the difference between preoperative and postoperative ROM for the 21 participants

Parameter	Control group ROM pre, post, improvement	3D group ROM pre, post, improvement
Flexion (Median [IQR])	90 [30], 110 [40], 10 [5, 27]	90 [10], 100 [30], 5 [5, 17]
Internal rotation (Median [IQR])	0 [10], 30 [30], 30 [20]	0 [5], 30 [20], 25 [20]
External rotation (Median [IQR])	20 [30], 0 [20], -20 [40]	10 [15], 0 [5], -17,5 [33,75]
Abduction (Median [IQR])	25 [20], 35 [30], 10 [5, 17]	30 [40], 35 [14], 10 [10]

**Table 3:** Clinical results showing primary and secondary outcomes for the 21 participants. The ROM is the difference between post-operative ROM and the normal values [20, 21]

Parameter	Control group	3D group	P value
Postoperative ROM compared to normal values (in degrees)			
Flexion (Mean ± SD)	-20 ± 19,0	-28,8 ± 21,2	0,28
Internal rotation (Median [IQR])	-15 [30]	-15 [20]	0,40
External rotation (Mean ± SD)	-14,3 ± 15,7	-5,4 ± 16,0	0,37
Abduction (Mean ± SD)	-6,7 ± 12,1	-4,2 ± 9,7	0,14
Radiographic improvement			
Southwick angle (Mean ± SD)	-15,6 ± 14,2	-20,1 ± 21,5	0,31
Questionnaires			
Physical PedsQL (Mean ± SD)^*^	84,4 ± 16,5	74,3 ± 15,6	0,09
Psychosocial PedsQL (Median [IQR]) ^*^	90 [3, 13]	91,7 [3, 28]	0,31
Total PedsQL (Median [IQR]) ^*^	89,1 [9, 23]	88,0 [4, 26]	0,21
HOOS-PS (Mean ± SD) ^*^	11,3 ± 12,3	15,3 ± 14,3	0,27
Surgery time			
Surgery time in minutes (Median [IQR])	156 [75]	147 [26]	0,25

^*^The scores of all questionnaires range from 0 to 100. In the PedsQL a lower score means more complaints, in the HOOS-PS a higher score means more complaints

Radiographic improvement, defined as the Southwick angle, was larger in the 3D group (-20,1 ± 21,5 vs. -15,6 ± 14,2 ), but not statistically significant.

Patient-reported clinical outcomes assessed with two questionnaires did not show significant differences between the two groups. On the physical PedsQL questionnaire patients had a mean score of 74,3 with a Standard Deviation of 15,6 in the 3D group and 84,4 ± 16,5 in the classic group. On the Psychosocial PedsQL questionnaire the median score was 91,7 with an Interquartile Range (IQR) of 28,3 compared to 90 [3, 13] and on the HOOS-PS the patients scored 15,3 ± 14,3 vs. 11,3 ± 12,3.

Duration of surgery was shorter within the 3D planned group (156 min with an IQR of 75 vs. 147 min with an IQR of 26), but not statistically significant. There was no learning curve found.

Follow up time was viewed as a potential confounder. Follow-up time for the classic Imhäuser group was longer than for the 3D planned group, because the patient in the classis Imhäuser group were operated before 2014, while the last patient in the 3D group was operated in 2019.

Since the questionnaires were sent later than the latest physical exam, two different follow up times were used: one for the physical exam and the other for the questionnaires. A linear regression test was used to assess follow up time as a potential confounder (Table 4). The result showed no statistically significant confounding effect.

**Table 4:** Correcting ROM and outcome of questionnaires for follow-up time as a confounder using linear regression

Variable	Coefficient	P value
Flexion	0,44	0,13
Internal rotation	0,35	0,12
External rotation	-0,21	0,38
Abduction	0,13	0,42
Physical PedsQL	0,15	0,08
Psychosocial PedsQL	0,06	0,55
Total PedsQL	0,09	0,29
HOOS-PS	-0,06	0,44

## Discussion

The main goal of the current study was to determine if a 3D preoperative planning and the use of 3D-printed surgical guides in Imhäuser osteotomy compared to classic Imhäuser osteotomy improved the Range of Motion of the hip after surgery. This study did not find any statistically significant differences between both techniques.

Improvement in ROM was chosen as primary outcome parameter, because the main goal of the surgery is to improve the ROM to increase mobility of the patient. Improvement of ROM was seen in both groups, with a slightly larger improvement in the 3D group, but no statistically significant difference between both groups was observed. Literature regarding improved clinical outcome using 3D surgical guides is scarce for SCFE patients, although in other surgical areas literature is available regarding the improved outcome when using surgical guides [24–26], suggesting a possible added value in SCFE patients. Based on our study we cannot draw a conclusion regarding the added value of surgical guides for improvement of ROM.

The radiographic improvement, defined as the Southwick angle, was slightly larger in the 3D group, but not statistically significant different between both groups. The Southwick angle preoperatively was slightly larger in the 3D group compared to the classic group, indicating a group of patients with a more severe slip, but the difference was not statistically significant. Cherkasskiy et al. [17] showed that the use of 3D patient specific models results in shorter OR times and less fluoroscopy dose while maintaining the same surgical results. Zakani et al. [18] performed the correction osteotomy on 3D-printed bones and found an improved accuracy of the guided group compared to the non-guided group. The guided surgery group also required significantly less drilling time and intraoperative X-rays. Zakani et al. performed their study in the laboratory, which is an optimal surrounding for performing the osteotomy. Different factors might explain the differences between their study and our results. In laboratory there is full exposure of the bones, therefore limited visibility through an incision is not a limiting factor. Furthermore, because the bones were based on the CT segmentation the surgical guides fit perfectly, this is in contrast to the situation in the OR, where the bones are never perfectly clean and do not perfectly match the CT segmentation.

The correct positioning of the surgical guide is of utmost importance to be able to accurately perform the osteotomy. Incorrect positioning might lead to deviations from the planned correction [27–30]. Possible causes might be the segmentation of the CT scan used for the design of the guide, the production of the surgical guides and the positioning of these guides. The cylindrical form of the femur makes it challenging to accurately position the surgical guide, despite the press-fit, especially in obese children and with limited visibility through the incision. The small differences in clinical outcome within the 3D group compared to the classic group might be explained by a suboptimal positioning of the guide.

Patient reported outcome, based on two different questionnaires, was not significantly different between both groups. Follow-up time was significantly different between both groups, but regression analysis showed no statistically significant effect on the questionnaires and ROM.

Though surgery time was not statistically significant different between the two groups, the 3D planned group showed a slightly shorter surgery time, which is in agreement with Cherkasskiy et al. [17]. Advantages of shorter surgery time are e.g. lower blood loss, less anesthesia and less costs [31].

Cherkasskiy et al. [17] also showed reduced radiation dose. We did not evaluate perioperative radiation dosage due to a lack of registry of perioperative fluoroscopy duration and radiation dosage of the patients. Radiation dosage was only registered in 9 of the included patients, all of whom where in the 3D planned group. Due to the lack of information regarding radiation dosage in the control group, and the lack of information on the average radiation dosage for a Imhäuser osteotomy in literature, it is not possible to assess the dosage reduction by using 3D planning and a surgical guide.

This study should be interpreted in light of its limitations. The results should be interpreted with the small sample size in mind. The surgery is performed only in patients with persistent complaints (pain, discomfort), or patients with severe limited ROM (lost internal rotation). Since the implementation of 3D planning in 2014, only 16 patients underwent an Imhäuser osteotomy, of which 14 were included in this study.

Because of the small number of patients undergoing this type of surgery, a power analysis was not done for this study, we just included all available patients.

Although the results of this study have not shown significant evidence of the added value of 3D preoperative planning and 3D-printed surgical guides in Imhäuser osteotomy compared to the classic Imhäuser osteotomy, the slightly larger radiographic improvement and shorter operation time, together with results from literature suggesting improved accuracy, support further research. This research should focus on the positioning of the surgical guide, because this might explain the differences between results in laboratory and clinical results.

## Data Availability

The datasets generated and/or analysed during the current study are not publicly available due to privacy reasons. The approval of the local ethics committee and the patients does not include sharing data. Data are however available from the authors upon reasonable request and with permission of the patients.

## Abbreviations

SCCFE: Slipped Capital Femoral Epiphysis
ROM: Range Of Motion
HOOS-PS: Hip disability and Osteoarthritis Outcome Score - Physical function Short form
PedsQL: Pediatric Quality of Life inventory
IQR: InterQuartile Range
SD: Standard Deviation
